# Transcriptomic analyses of gastrointestinal function in the "dwarf" and "medium" forms of *Sthenoteuthis oualaniensis* during sexual maturation

**DOI:** 10.1371/journal.pone.0199053

**Published:** 2018-06-13

**Authors:** Lulu Yan, Chao Zhao, Jun Zhang, Lihua Qiu, Zuozhi Chen

**Affiliations:** 1 South China Sea Fisheries Research Institute, Chinese Academy of Fishery Sciences, Guangzhou, P. R. China; 2 Key Laboratory of South China Sea Fishery Resources Exploitation & Utilization, Ministry of Agriculture, Guangzhou, P. R. China; 3 Key Laboratory of Open-Sea Fishery Development, Ministry of Agriculture, Guangzhou, P. R. China; 4 Key Laboratory of Aquatic Genomics, Ministry of Agriculture, Beijing, P. R. China; Xiamen University, CHINA

## Abstract

*Sthenoteuthis oualaniensis* (SA) is an important squid species in the South China Sea. Based on SA samples collected in 2016, SA was divided into the “dwarf” form (DF) and “medium” form (MF). To understand the changes in gastrointestinal function in SA during sexual maturation, we undertook transcriptomic analyses of the stomach and intestine tissues of the mature and immature DF and MF of SA using the deep-sequencing platform Illumina HiSeq™. We exploited a high-throughput method to delineate differentially expressed genes (DEGs) in the DF and MF of SA. A total of 135464 unigenes (68627 unigenes of the DG and 66837 unigenes of the MF) were generated. We identified 7965 and 4051 relative DEGs in the intestine and stomach tissues of the mature DF of SA compared with those of the immature DF of SA; and 22138 and 18460 DEGs in the intestine and stomach of the mature MF of SA compared with those of the immature MF of SA. Gastrointestinal function related to the metabolism of lipids, amino acids, glucose, and energy were changed in SA during sexual maturation. This work is the first to identify a set of genes associated with gastrointestinal function during sexual maturation in SA.

## Introduction

*Sthenoteuthis oualaniensis* (SA; “purpleback flying squid”) is distributed widely along the equator throughout the Indo-Pacific region (40°N to 40°S) [1, 2]. SA is considered to be the most abundant large squid of commercial importance in the region [3]. Within this wide distribution, SA is also known to have a wide ecological amplitude and complex population structure [1]. Two major forms of this species occur in the southern South China Sea based on their morphological characteristics: ‘dwarf’ and ‘medium’. The larger medium form (MF) exhibits a dorsal photophore whereas the dwarf form (DF) does not. [3, 4]. These two forms are also distinct in terms of genetics and morphology. The population structure, sex ratios, sexual maturity, and mortality of the MF and DF have been studied [3, 5].

The stage of sexual maturity in SA can be divided into І to V using Lipinski's maturity scales for male and female squids [6]. The mantle length (ML) of the sexually mature female MF is 190–250 mm, whereas that of the male MF is 120–150 mm. The ML of the sexually mature DF is 90–120 mm, which is shorter than that of the MF. In general, amphipods, euphausiids, and fish larvae are the primary food sources for juvenile squids. In contrast, the adults eat mainly myctophids but can show cannibalistic behavior [1, 7, 8]. Based on data collected by fisheries surveys, the relationship between a predator and prey is determined by body size. Crustacea and larvae squid are the main foods of SA with a ML of 40–100 mm. The proportion of crustacea eaten by SA with a ML 100–150 mm is low. SA with a ML of 150 mm mainly eat myctophids [9]. An increase in the ML of SA leads to changes in feeding habits. Therefore, the ML or body size has a close connection with sexual maturity and feeding habits. In land-dwelling and aquatic mammals, sexual maturity is, in general, affected by the structures of amino acids, lipids and carbohydrates [10, 11]. Moreover, developmental stages elicit changes in dietary patterns, which may influence the absorption and digestion functions of the stomach and intestines. Studies have shown that the expression of genes related to the synthesis of steroid hormones, crustacean juvenile hormone and vitellogenin were changed in the hepatopancreas during ovarian development in *Portunus trituberculatus* [12]. Gastrointestinal function and reproductive systems are closely situated and intertwined, which optimizes the potential for the transfer of nutrients and energy to developing gametes [13]. Studies have shown that nutrients from the lume are transported to the basal lamina by intestinal cells, and then hemocytes carry them to surrounding gonad tissue in scallops [14]. Therefore, understanding the relationship between gastrointestinal function and sexual maturity in relation to gene expression is important.

To explore the genes associated with gastrointestinal metabolism as well as their relationships to sexual maturity, RNA-sequencing (RNA-Seq) technology was used to estimate the expression of genes associated with gastrointestinal function in the DF and MF of SA during maturation. The results of our study could enhance understanding of the genes associated with gastrointestinal function and their roles in the different stages of sexual maturity of SA. It can also be helpful for the research of feeding habits and digestion of squids, which are associated with the growth and distribution of fish populations.

## Materials and methods

### Ethics statement

SA is not an endangered or protected species, and there is no requirement for permission to undertake experiments involving this species in China. The authority responsible for the sea in each location issued the permission.

### Experimental design

The DF and MF of SA were procured from the South China Sea (10°33"N, 115°53"E) on 20 November 2016. They were classified into “mature” SA (maturity stages III–V) and “immature” SA (maturity stages I and II). Intestinal and stomach tissues were sampled from three mature DF SAs, three immature DF SAs, three mature MF SAs, and three immature MF SAs, respectively. Twenty-four samples (8 groups × 3 parallels) were collected from SA: Dwarf form-Undevelopmental-Intestine (DUI), Dwarf form-Developmental-Intestine (DDI), Dwarf form-Undevelopmental-Stomach (DUS), Dwarf form-Developmental-Stomach (DDS), Middle form-Undevelopmental-Intestine (MUI), Middle form-Developmental-Intestine (MDI), Middle form-Undevelopmental-Stomach (MUS) and Middle form-Developmental-Stomach (MDS). All sample collections were undertaken in RNAlater™ solution (Invitrogen, Carlsbad, CA, USA).

### RNA extraction and preparation of sequencing libraries for transcriptomic analyses

Total RNA was extracted using a mirVana™ miRNA Isolation kit (Thermo Scientific, Waltham, MA, USA) and treated with RNase-free DNase I. RNA integrity was assessed using a 2100 Bioanalyzer (Agilent Technologies, Santa Clara, CA, USA). Samples with a RNA Integrity Number ≥7 were used for subsequent analyses. Sequencing libraries were generated using a TruSeq™ Stranded mRNA Sample Prep kit (Illumina, San Diego, CA, USA) according to manufacturer instructions. Then, the paired-end RNA-Seq library was sequenced with a sequencing platform (read length = 2 × 150 bp; HiSeq X Ten; Illumina). The 24 samples were run separately and separate libraries were generated.

### Analyses of sequencing data and functional annotation

Clean reads were used to undertake *de novo* transcriptome assembly with Trinity software [15]. Two datasets of unigenes were assembled from the reads of DF (DF dataset, assembled from the reads of DUI, DDI, DUS, and DDS groups) and MF (MF dataset, assembled from MUI, MDI, MUS and MDS groups) of SA. All assembled unigenes with an E-value <1.0 × 10^−5^ were matched against public databases [National Center for Biotechnology Information (NCBI) protein non-redundant (nr), SWISS-PROT, Eukaryotic Orthologous Groups (KOG), Gene Ontology (GO), Kyoto Encyclopedia of Genes and Genomes (KEGG), malTFDB].

### Analyses of differential expression and functional enrichment

A differential expression analysis for common tissue (intestine or stomach) during gonadal development of the DF and MF of SA was undertaken to identify differential-expression patterns for four pairwise comparisons: DUI *vs*. DDI (PC1), DUS *vs*. DDS (PC2), MUI *vs*. MDI (PC3) and MUS *vs*. MDS (PC4). The fragments per kilobase of transcript per million mapped reads (FPKM) value was used to identify differentially expressed genes (DEGs) between treatment and control samples. DEGs were identified by EDSeq (http://bioconductor.org/packages/release/bioc/html/DESeq.html). *P* < 0.05 and an absolute value of log_2_ fold change ≥1 were set as the criteria for significant differential expression. GO and KEGG pathway enrichment analyses were used for functional categorization of DEGs. GO enrichment analyses were done using Blast2GO. TopGO (www.bioconductor.org/packages/2.11/bioc/html/topGO.html) was employed to draw directed acyclic graphs (DAGs) of the DEGs based on GO enrichment.

### Expression according to quantitative reverse transcription-polymerase chain reaction (qRT-PCR)

Twelve annotated genes (six genes for the DF and six genes for the MF) were analyzed further by qRT-PCR. Total RNA was obtained using TRIzol™ 1 Reagent (Thermo Scientific). First-strand cDNA was synthesized from 950 ng of total RNA using a PrimeScript™ RT Reagent kit (TaKaRa Biotechnology, Dalian, China) in accordance with manufacturer protocols. cDNA-specific primers were designed using AlleleID v6.0 (Table 1). Cytochrome B (CYTB) and elongation factor 1-alpha (EF1A) were chosen as the reference genes for the DF. Glyceraldehyde 3-phosphate dehydrogenase (GAPDH) and EF1A were chosen as the reference genes for the MF. qRT-PCR was undertaken using a LightCycler^®^ 480 system employing TB Green™ Premix Ex Taq™ II (Tli RNase H Plus) (TaKaRa Biotechnology). Amplifications were done in a 96-well microtiter plate in a final reaction volume of 20 μL containing 10 μL of SYBR Premix Ex Taq II (Tli RNaseH Plus) (2×), 0.8 μL (each) of gene-specific forward and reverse primers (10 μM), 6.4 μL of RNase-free water, and 2.0 μL of cDNA (<100 ng). PCR conditions were 95°C for 30 s followed by 40 cycles of 95°C for 5 s, 58°C for 30 s and 72°C for 30 s. Formation of a single product was confirmed for all genes tested using melting curves.

**Table 1:** Primer sequences for amplification of target and reference genes in two dwarf-form and medium-form *Sthenoteuthis oualaniensis*.

Gene ID	Gene name	Primer sequence
**Target gene in DF**		
MSRE	Macrophage scavenger receptor	GCAGGATTGGGAGGAAAGCATTAC
		CGGCACACTACTTTGGCATCG
ELOVL6	Elongation of very long chain fatty acids protein 6	TGCTGTCATTCTTGTGGTCATTCC
		CCTGGCTCGGGCTTTCATCC
ELOVL6	Guanine nucleotide-binding protein subunit alpha-12	TCCCTGCGTTCAACAAAGACC
		CGATTGCTGTGGTGAAATGATGG
CBP	Chitin binding beak protein	ACATTACAGAATTGCGACTTGCC
		GTTGTTGTGGACGGATTGAGC
VAMP	Vesicle-associated membrane protein	TGCCGTTGAAGTCAGAAAGAAAGC
		TGTTGGTGTGGGTGGTGTTGTC
MMACHC	Methylmalonic aciduria and homocystinuria type C protein homolog	GCAAGAATCCTAGTCCAGACAGC
		GGAGGCGGTGTTGGTTTCAG
**Reference gene in DF**		
CYTB	Cytochrome b	TGACCGTATCGCCGTTGTTG
		CAGTGCCCATTGCCATTTGC
EF1A	Elongation factor 1-alpha	CCTGGCTGTATTCTGCGGTATCC
		GGTTGTGGCTGCTGGTGTTGG
**Target gene in MF**		
AMI	Aminopeptidase	GTCTTCTTAGTTGGAAAGGGTATC
		GCACGGGAGGTCACAATC
NS2	Asparaginyl-tRNA synthetase	AACTGCGAGATACTATCCAGACAC
		TGACCAACGACTTCCCAATAATCC
Apop1	Apoptogenic protein 1	CTGATGAGGTCGGATATGCTTCG
		GGACTGGTCGCAAGTTGGAG
Ctrb	Chymotrypsin B	GCAGATAGCAGTCCCATTCC
		AAGGTCATCCATAACGCAGTG
Crb	Protein crumbs	CGTATGGCTCCTACAACTGC
		GAGGTGTGCGTCTGGTTC
Dhc	Dynein heavy chain	CCTTCTACTCATTCGTTGCTGGTG
		GCGTTCGGTTGTTGCTCTCC
**Reference gene in MF**		
GAPDH	Glyceraldehyde-3-phosphate dehydrogenase	GTCAACCACCACCAATACAAGAAG
		CCTTAGCGGCACCAGTTCC
EF1A	Elongation factor 1-alpha	GCGGTAGCAGCAGCATCATC
		TCACTCCCATCTATCAGGTCTTCC

## Results

### Sequencing and de novo assembly

**Fig 1:**
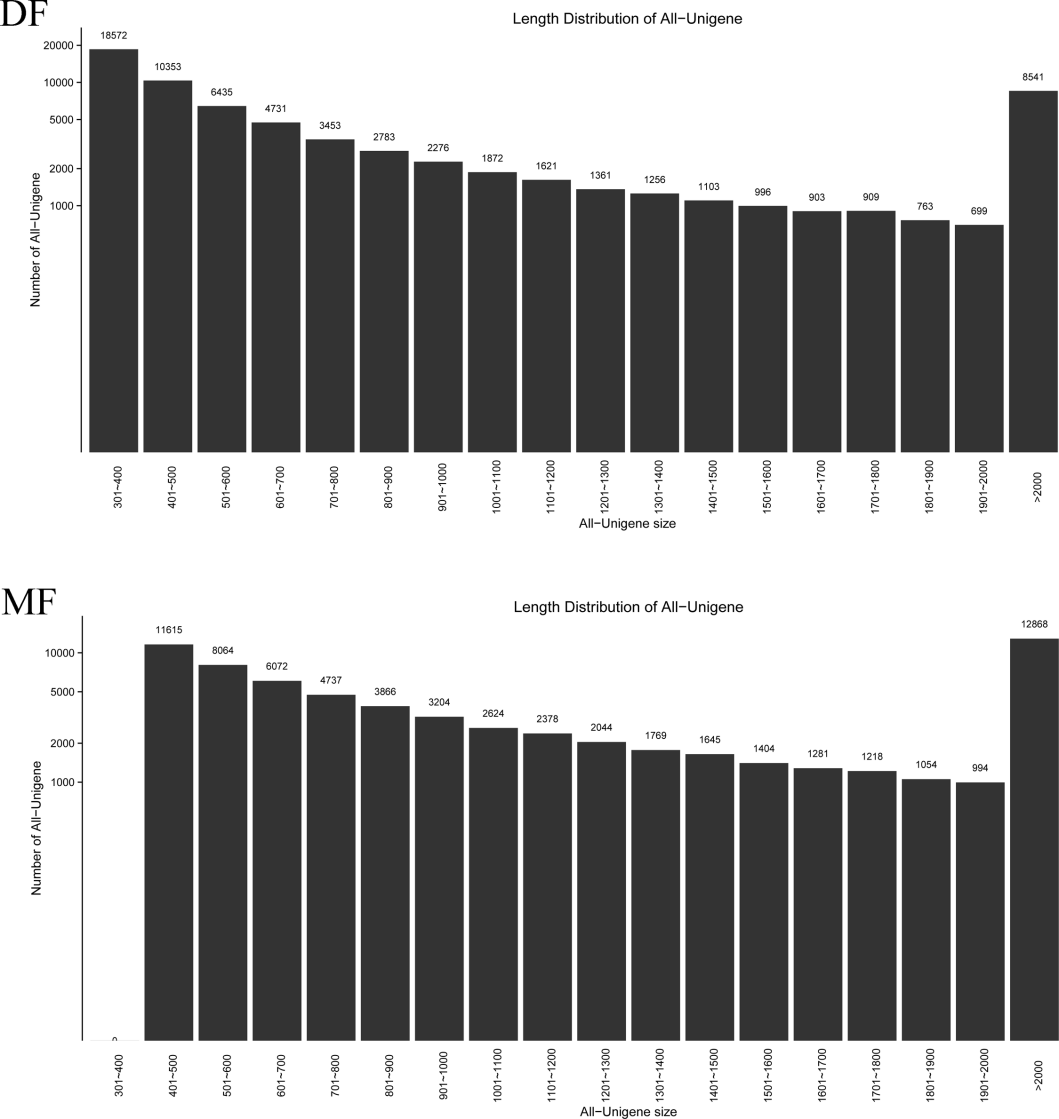
Distribution of unigenes in the DF and MF of SA. The horizontal axis shows the length interval of the size of unigenes. The longitudinal axis shows the number of unigenes.

A total of 599.38 million raw reads were produced from the DF data set, whereas 1585.33 million raw reads were produced from the MF data set (S1 Table). After quality trimming and adapter clipping, 570.63 million clean reads of the DF data set and 556.04 million clean reads of the MF data set were retained. Following removal of redundant sequences, 135464 unigenes (68627 unigenes of the DF and 66837 unigenes of the MF) were generated from both data sets. An average length of 1194 bp and 95116 unigenes were longer than 500 bp. The distribution of unigene length is shown in Fig 1, and varied from 300 bp to >2000 bp. All sequences with the raw reads were submitted to the NCBI Sequence Read Archive under the accession number PRJNA430301.

### Functional annotation and analyses of transcription factors (TFs)

**Fig 2:**
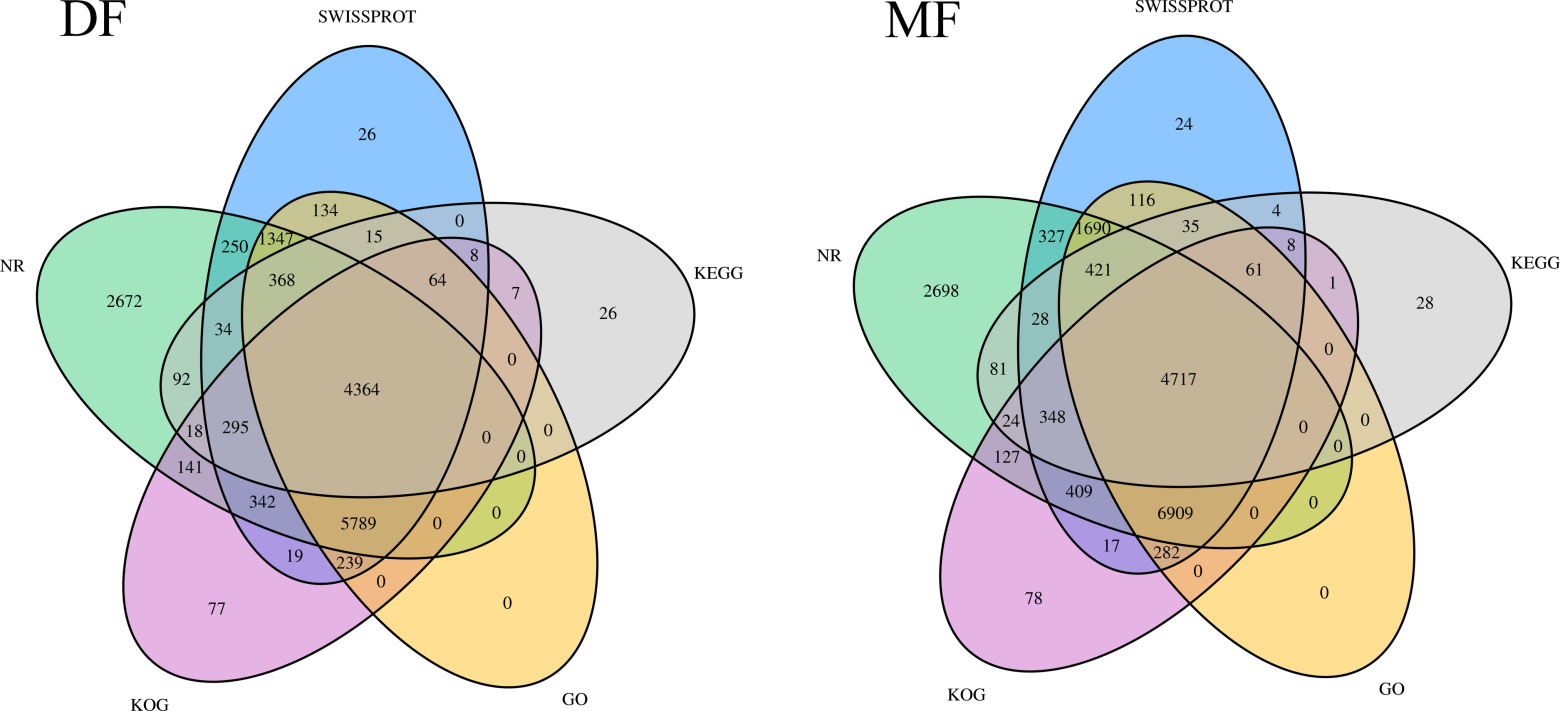
Venn diagram illustrating the number of annotated unigenes in the DF and MF of SA.

The 135464 unigenes were annotated. Of these unigenes, 33491 (24.75%) showed significant (*P*<0.05) BLASTx matches in the nr database and 28690 (21.21%) showed significant (*P*<0.05) matches in the Swiss-Prot database. A Venn diagram of the annotation results of DF and MF data sets is shown in Fig 2. In summary, a total of 16327 unigenes (23.79%) of the DF and 18433 unigenes (27.58%) of the MF of SA were annotated by at least one of the five databases used.

A total of 11363 unigenes of the DF and 12981 unigenes of the MF were annotated in KOG and were both categorized into 26 groups. The ‘various KOG categories, general function prediction only’ (R, 28.73% of the DF and 30.38% of the MF) was the most highly represented, followed by the ‘signal transduction mechanisms’ (T, 10.25% of the DF and 13.50% of the MF).

**Fig 3:**
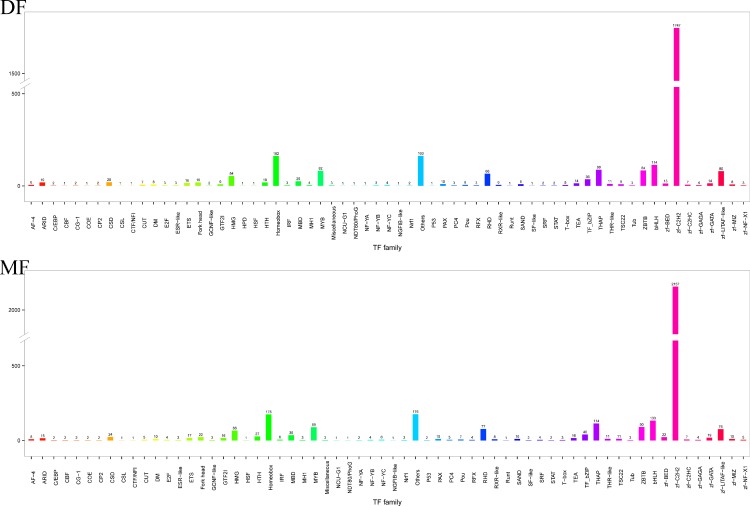
The number of unigenes annotated to TF families. The horizontal axis shows the name of the TF family. The longitudinal axis shows the number of unigenes classified into different TF families.

malTFDB is a database for the TFs of animals. These TFs are classified into 70 families based on their DNA-binding domain. Of the 135464 defined unigenes, 6893 unigenes (3302 unigenes of the DF and 3591 unigenes of the MF) were annotated and classified into 65 TF families. Among the various TF families, the zf-C_2_H_2_ family had the highest number of unigenes (1747 unigenes of the DF and 2157 unigenes of the MF), and the “other TF family” had the second largest number of unigenes (163 unigenes of the DF and 176 unigenes of the MF), followed by the Homeobox TF family (162 unigenes of the DF and 175 unigenes of the MF). One unigene of the DF was classified into CBF, CSL and “other” 13 TF families. One unigene of the MF was classified into NDT80/PhoG, HSF and “other” 7 TF families (Fig 3).

### DEG analyses

**Fig 4:**
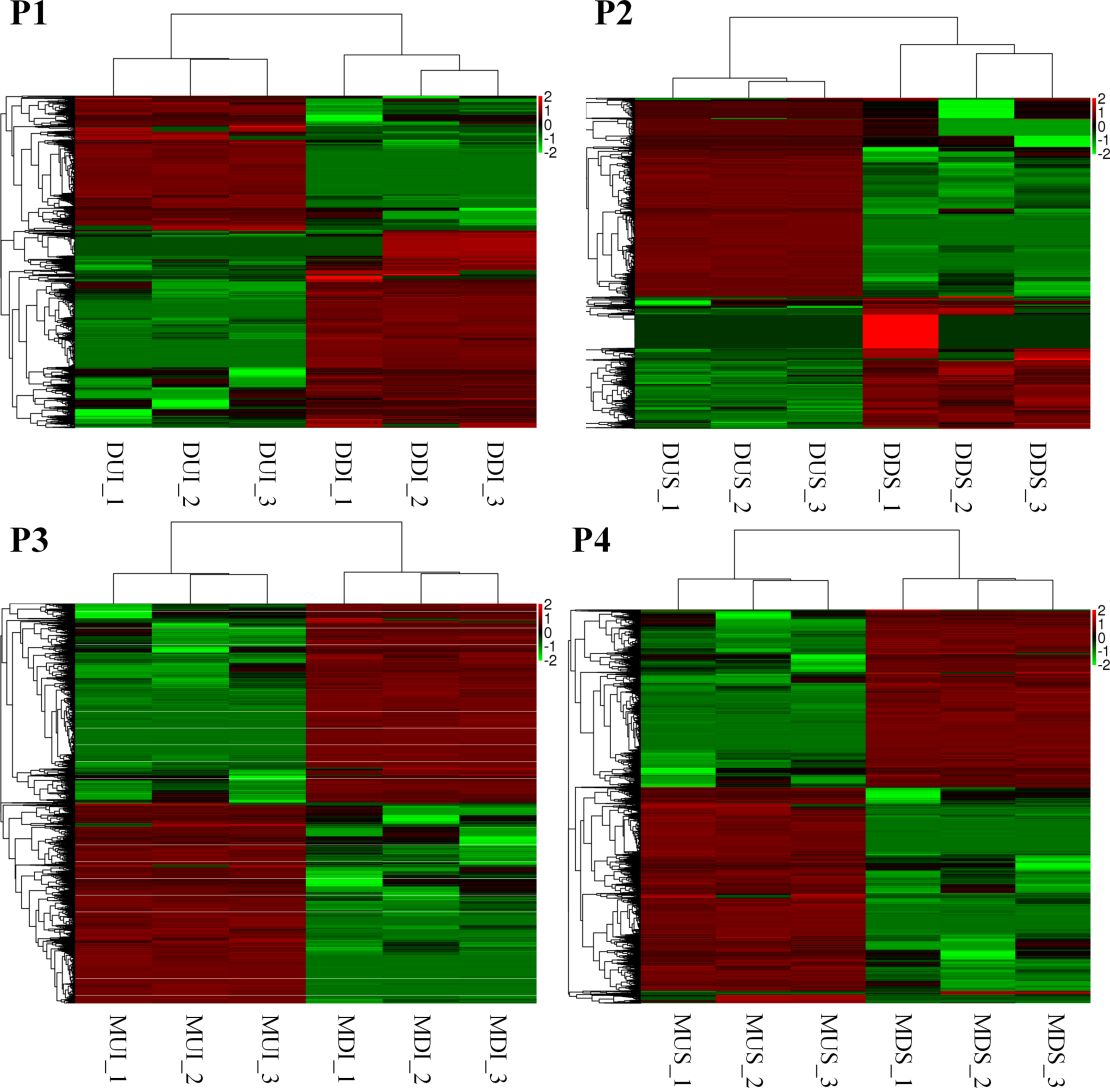
Cluster analyses of DEGs of SA. Green represents lower expression, and red represents higher expression. Each column represents a comparison: PC1, PC2, PC3 and PC4. Each row represents a gene.

In total, 7965, 4051, 22138 and 18460 DEGs were identified for PC1, PC2, PC3 and PC4, respectively (Fig 4), with a filtering criterion of the absolute value of log_2_ fold change ≥1 and *P* < 0.05. In the DF assembly data set, 1046 DEGs were present in PC1 and PC2, whereas 6919 and 3005 DEGs were PC1-specific and PC2-specific, respectively. In the MF assembly data set, 9239 DEGs were present in PC3 and PC4, whereas 12899 and 9211 DEGs were PC3-specific and PC4-specific, respectively.

### GO annotations and pathway analyses of DEGs

**Fig 5:**
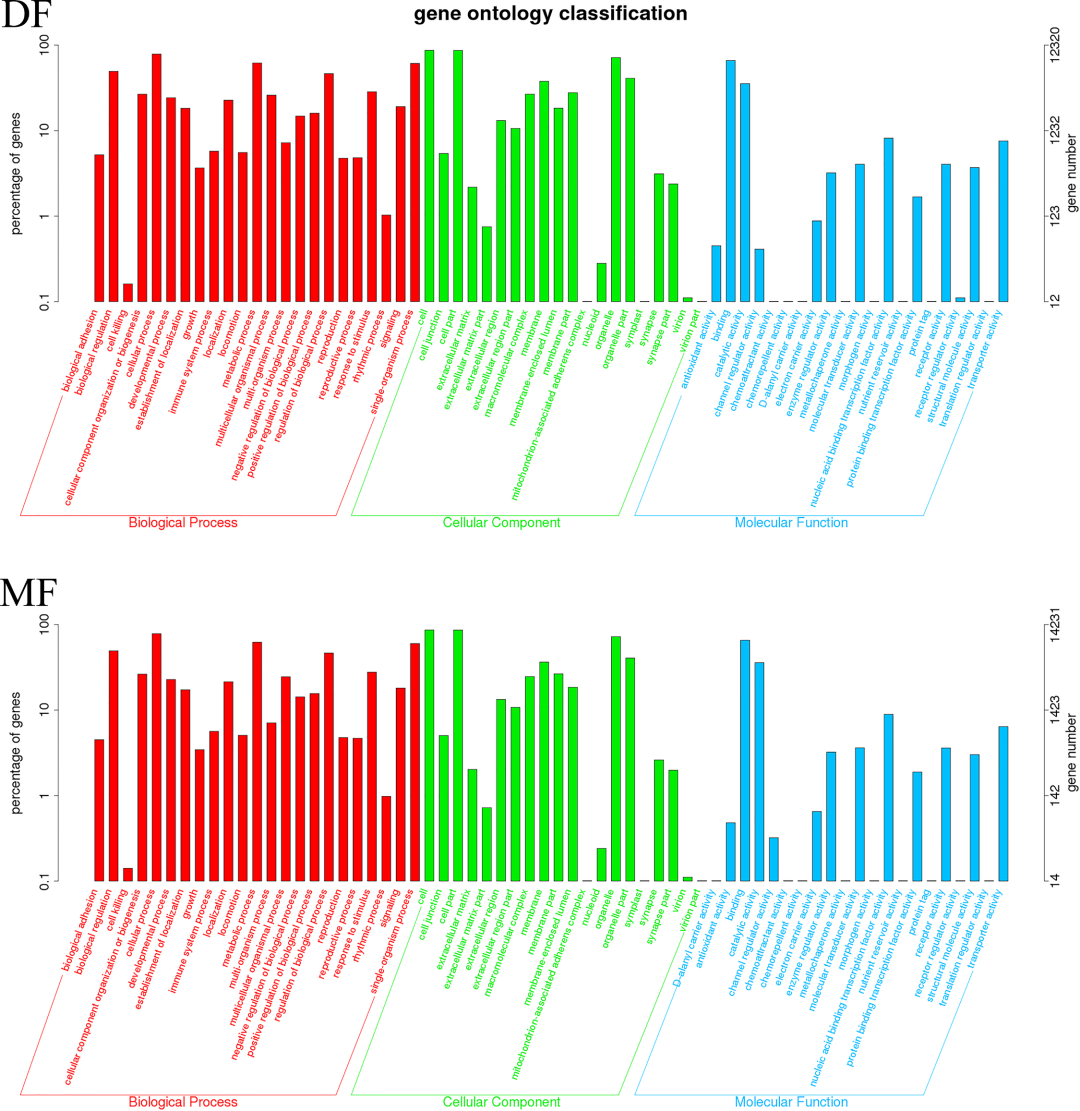
The number and percentage of GO annotated unigenes.

**Fig 6:**
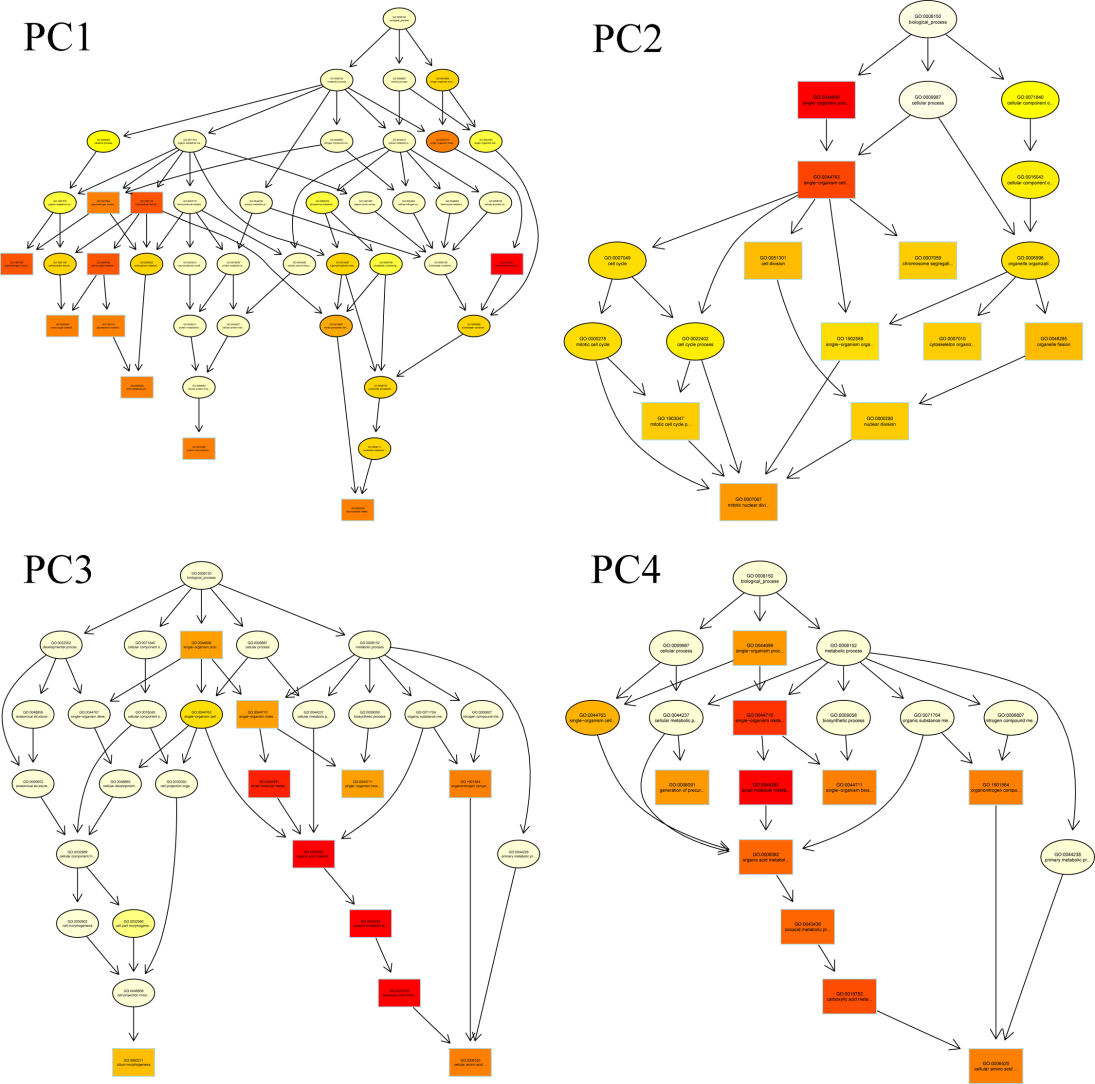
Directed acyclic graphs of PC1 to PC4. The branch shows the containment relationships of GO terms. The GO term with a deeper color represents more significant enrichment.

All GO assignments were assigned to three categories: molecular function, biological process, and cellular component (Fig 5). The significantly enriched biological process terms were worthy of attention. Four directed acyclic graphs (DAGs) from PC1 to PC4 were used to model causal relationships between enriched biological process terms. In such models, some GO terms [Chitin metabolic process (GO: 0006030) of PC1; Mitotic nuclear division (GO: 0007067) of PC2; Cilium assembly (GO: 0060271) of PC3; Cellular amino acid metabolic process (GO: 0006520) of PC4] were found to be enriched in the DF or the MF of SA during gonadal development (Fig 6). These data indicated that these biological processes may have crucial roles in the differential gonadal development between the intestine and stomach tissues of SA.

Besides GO analyses, the DEGs were mapped to various pathways based on KEGG analyses. The DEGs of PC1, PC2, PC3 and PC4 were mapped to 302, 328, 352 and 353 pathways as described in KEGG. All the significantly enriched KEGG pathways (*P*_adjusted_ < 0.05) corresponding to the DEGs detected in PC1–PC4 are shown in Table 2. These pathways included those related to: lipid metabolism-Steroid hormone biosynthesis (enriched pathway of PC1, ko00140) and alpha-Linolenic acid metabolism (enriched pathway of PC4, ko00592); amino acid metabolism-Phenylalanine, tyrosine and tryptophan biosynthesis (ko00400) and Phenylalanine metabolism (ko00360); carbohydrate metabolism-Glycolysis/Gluconeogenesis (ko00010) and Glycosaminoglycan degradation (ko00531); vitamin metabolism-Vitamin digestion and absorption (ko04977), Riboflavin metabolism (ko00740), and energy metabolism- Oxidative phosphorylation (ko00190).

**Table 2:** Significantly enriched KEGG pathways from PC1 to PC4.

ID	Term	List Hits	*P*	*P*_adjusted_
**PC1**				
ko00190	Oxidative phosphorylation	38	7.88 × 10^−8^	4.11 × 10^−6^
ko04974	Protein digestion and absorption	22	3.33 × 10^−5^	0.001157
ko04151	PI3K-Akt signaling pathway	34	5.38 × 10^−5^	0.0014218
ko00400	Phenylalanine, tyrosine and tryptophan biosynthesis	3	5.45 × 10^−5^	0.0014218
ko00520	Amino sugar and nucleotide sugar metabolism	14	0.000178	0.00398
ko00360	Phenylalanine metabolism	5	0.0002336	0.0048739
ko04512	ECM-receptor interaction	17	0.000256	0.0050089
ko01210	2-Oxocarboxylic acid metabolism	6	0.0003053	0.0056217
ko04510	Focal adhesion	28	0.0004389	0.0076313
ko00350	Tyrosine metabolism	6	0.0009684	0.0159524
ko04260	Cardiac muscle contraction	16	0.0013068	0.0198084
ko05130	Pathogenic Escherichia coli infection	13	0.0016309	0.0232032
ko05418	Fluid shear stress and atherosclerosis	19	0.0017124	0.023303
ko04015	Rap1 signaling pathway	23	0.0020369	0.0265645
ko04977	Vitamin digestion and absorption	7	0.0024202	0.0303006
ko04912	GnRH signaling pathway	16	0.0027263	0.0328206
ko00140	Steroid hormone biosynthesis	6	0.0041114	0.0459593
**PC2**				
ko04111	Cell cycle—yeast	22	2.98 × 10^−7^	9.91 × 10^−5^
ko03030	DNA replication	15	5.41 × 10^−6^	0.0009011
ko04810	Regulation of actin cytoskeleton	26	0.0001727	0.0115012
ko04110	Cell cycle	24	0.0002625	0.014569
ko00520	Amino sugar and nucleotide sugar metabolism	15	0.0005688	0.0236768
ko04723	Retrograde endocannabinoid signaling	22	0.001057	0.0364929
**PC3**				
ko00300	Lysine biosynthesis	2	0	0
ko00480	Glutathione metabolism	34	0.0001278	0.0090511
**PC4**				
ko00190	Oxidative phosphorylation	67	2.88 × 10^−5^	0.0038881
ko04145	Phagosome	58	7.62 × 10^−5^	0.0068348
ko00010	Glycolysis / Gluconeogenesis	35	0.000219	0.0131012
ko04142	Lysosome	68	0.0002565	0.0131532
ko00360	Phenylalanine metabolism	12	0.0009598	0.036489
ko00531	Glycosaminoglycan degradation	18	0.0010164	0.036489
ko00592	alpha-Linolenic acid metabolism	10	0.0012188	0.0392797
ko04932	Non-alcoholic fatty liver disease	60	0.001313	0.0392797

### Validation by qRT-PCR

**Fig 7:**
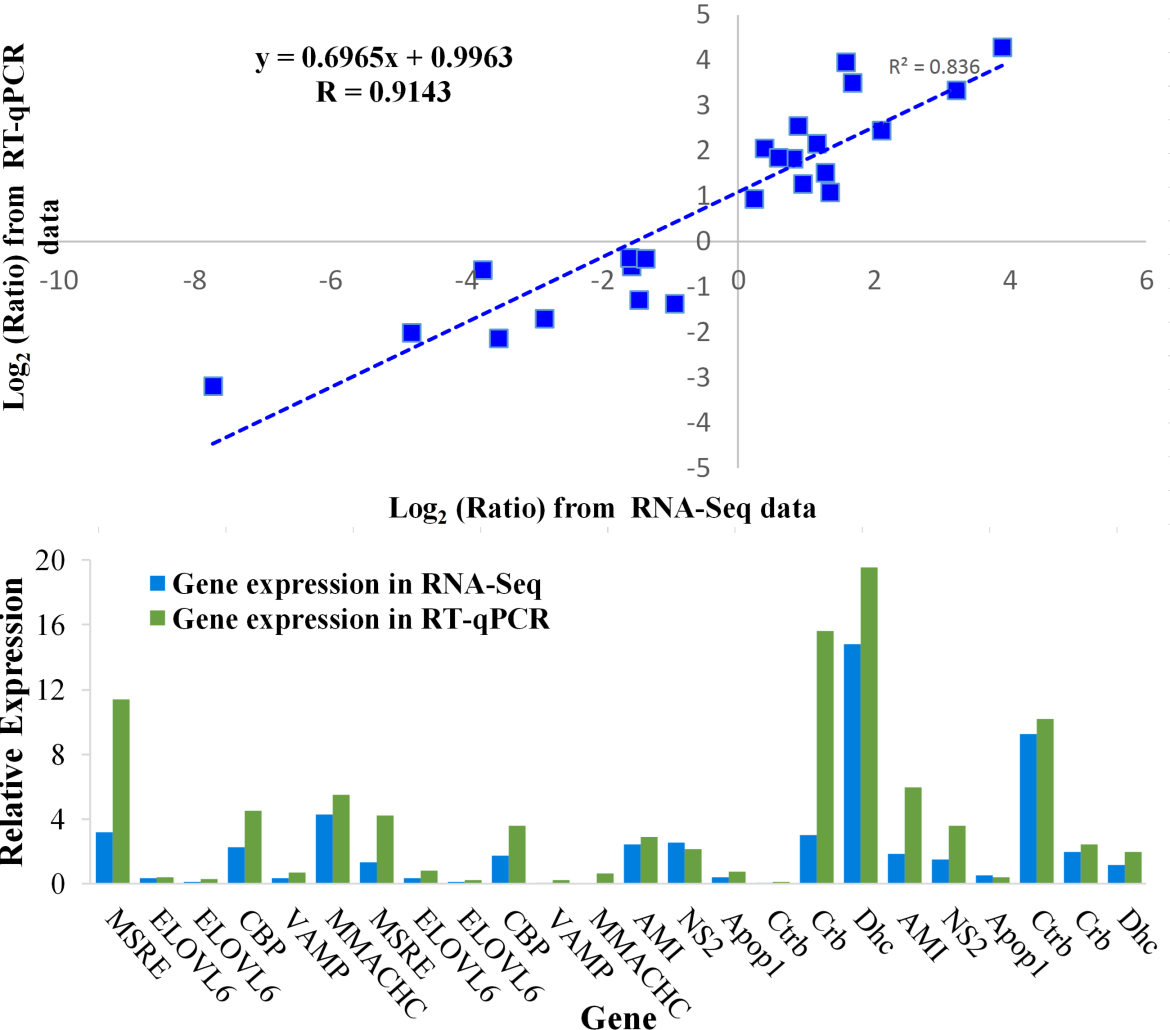
Linear regression analysis and column graphs of the gene expression between RNA-Seq sequencing and qRT-PCR.

To validate the accuracy and reproducibility of expression profiles, qRT-PCR was done to measure the expression of 12 randomly selected unigenes. Expression of these genes from PC1 to PC4 was measured by qRT-PCR and compared with that from RNA-Seq. These genes showed high correlation with RNA-Seq in the DF and MF of SA (R = 0.9143) (Fig 7). Hence, qRT-PCR confirmed our RNA-Seq data, suggesting the reliability and accuracy of our high-throughput analyses.

## Discussion

We employed sequencing technology to compare the transcriptome of common tissues (intestine and stomach) between the mature and immature DFs and MFs of SA. A set of DEGs was found which suggested that gonadal development can cause a dramatic change in the intestines and stomachs of the DF and MF of SA. This work is the first attempt to identify the genes essential to the function of the intestine and stomach during sexual maturation. We employed RNA-Seq to measure the expression of the genes and pathways related to the metabolism of lipids, amino acids, glucose, and energy.

### Lipid metabolism

Lipids are necessary for energy homeostasis, reproductive physiology, and other aspects of cellular biology [16]. Sexual maturity can lead to changes in the diet of SA [1]. Changes in dietary fatty acids can influence lipid metabolism directly, such as the transport/uptake of lipids, catabolism of fatty acids, and lipogenesis [17]. The DEGs were found to be involved in five lipid-metabolic pathways: steroid hormone biosynthesis (ko00140, enriched pathway of PC1); retrograde endocannabinoid signaling (ko04723, enriched pathway of PC2); arachidonic acid metabolism (ko00590, enriched pathway of PC3); alpha-Linolenic acid metabolism (ko00592, enriched pathway of PC4) and non-alcoholic fatty liver disease (ko04932, enriched pathway of PC4).

Interestingly, of these five pathways, steroid hormone biosynthesis (ko00140) and alpha-Linolenic acid metabolism (ko00592) have a close relationship with oocyte development and sexual maturity [18, 19]. Steroid hormones (e.g., 17β-estradiol, progesterone) can accelerate the maturation process in grey mullet fish through regulation of exogenous hormones in captivity [18]. In humans, 17β-estradiol and progesterone are responsible for controlling the female sexual cycle [20]. Also, six DEGs of PC1 were significantly enriched in this pathway, suggesting that these DEGs of the steroid hormone biosynthesis pathway may play an active part in the sexual maturation of the DF of SA. Increased expression of delta-6 desaturase (D6D) in the alpha—Linolenic acid metabolism pathway was identified in PC4. D6D is the first and rate-limiting enzyme for highly unsaturated fatty acid (HUFA) synthesis, which converts C18:2n6 and C18:3n3 to C18:3n6 and C18:4n3, respectively [21]. Studies have shown that HUFAs have a positive role in ovarian maturation, as well as the reproductive performance of crabs, shrimps and other aquatic animals [22]. We infer that specific fatty acids are required during the ovarian development of the MF of SA. In summary, sexual maturity may promote lipid metabolism in the intestine and stomach, and lipid metabolism may have an active role in the sexual maturation of the DF and MF of SA.

### Metabolism of amino acids

Studies have shown that several amino acids regulate key metabolic pathways which are necessary for the maintenance, growth, reproduction, and immune response in aquatic and terrestrial animals [23]. Four significantly enriched pathways related to amino-acid metabolism were identified in the intestine and stomach during sexual maturation of the DF and MF of SA: phenylalanine, tyrosine and tryptophan biosynthesis (ko00400); phenylalanine metabolism (ko00360); tyrosine metabolism (ko00350); lysine biosynthesis (ko00300).

Of these pathways, the phenylalanine metabolism pathway was found in all four pairwise comparisons, particularly in PC1 and PC4 (*p*_adjusted_ < 0.05). Except for protein synthesis, L-phenylalanine is converted to tyrosine and phenylpyruvate by phenylalanine hydroxylase and aminotransferase, respectively [24, 25]. Surprisingly, expression of phenylalanine hydroxylase and aminotransferase in the pathway of phenylalanine metabolism was down-regulated in the intestine and stomach of mature SA compared with immature SA. We suspect that most L-phenylalanine was used for the protein synthesis needed for sexual maturity, rather than being used for hydroxylation and transamination. Indeed, the protein-digestion and absorption pathway was activated in mature SA. Moreover, in this pathway, expression of L-type amino acid transporter (LAT) 1 was upregulated in the intestine of mature SA. LAT1 transports large neutral-pH amino acids (e.g., phenylalanine, tyrosine) into intestinal epithelial cells [26]. Then, these amino acids are released into the blood *via* multiple basolateral amino-acid transporters. Therefore, we infer that the amino-acid metabolism in the intestine and stomach of the DF and MF of SA was affected by sexual maturation, and that most amino acids were absorbed into blood to meet the needs of gonadal development.

### Glycolytic pathway

Glucose is a major source of cellular energy, and can be converted to pyruvate *via* the glycolytic pathway and its enzymes [27, 28]. Among these enzymes, fructose-1, 6-bisphosphate aldolases (FBA) and GAPDH are glycolytic proteins. FBA catalyzes beta-D-Fructose 1,6-bisphosphate to glycerone phosphate and D-glyceraldehyde 3-phosphate, which can be converted to 3-phospho-D-glyceroyl phosphate, the reduced form of nicotinamide adenine dinucleotide phosphate (NADPH), NADH and H^+^ by GAPDH. In the present study, DEGs in PC1 to PC4 were enriched in Glycolysis (ko00010). Moreover, expression of *FBA* and *GAPDH* was down-regulated in the intestine of mature SA compared with immature SA. We infer that the glycolytic pathway in the intestine of SA may be affected (and even inhibited) by sexual maturation. This may be due to an interrelation between dietary nutrients and sexual maturation. Simultaneously, they influence glucose metabolism in the intestine. Studies have shown that the activity of the key enzymes in glycolysis are influenced significantly by diet in fish [29, 30], findings that are similar to our results.

### Energy metabolism

Lipids, amino acids and carbohydrates as the main energy resources to be metabolized, coupled with metabolite dehydrogenation and reduction of NAD^+^ or flavin adenine dinucleotide [31]. Finally, H^+^ and electrons are transmitted to oxygen with the release a lot of energy. In the present study, 28, 28, 22 and 36 upregulated genes of PC1, PC2, PC3 and PC4, respectively, were enriched in oxidative phosphorylation (ko00190), suggesting that the latter was activated in the intestine and stomach during the sexual maturation of SA. In humans, increased energy expenditure in teenagers is observed during puberty and sexual maturation to reduce body fat and maintain optimal body weight [32]. We can infer that oxidative phosphorylation is needed to provide energy from the intestine and stomach of SA to promote maturation.

## Conclusions

This was the first study of the changes in gastrointestinal function in the DF and MF of SA during maturation using next-generation sequencing technology. A total of 965, 4051, 22138 and 18460 DEGs were altered significantly for PC1, PC2, PC3 and PC4, respectively. Several upregulated and downregulated genes involved in the metabolism of lipids, amino acids, glycogen, and energy were identified. We inferred that sexual maturity may promote lipid metabolism, affect the glycolytic pathway, and absorb energy *via* oxidative phosphorylation in the intestine and stomach of SA. These results highlight a complex network of nutritional and energy-based metabolic pathways in the intestine and stomach of the DF and MF of SA, and could form the basis of future research.

## Supporting information

S1 Table Production and filtering of short reads expressed using Illumina. (XLSX)
